# Diversity and Seasonal Dynamics of an Assemblage of Sarcophagid Diptera in a Gradient of Urbanization

**DOI:** 10.1673/031.011.9101

**Published:** 2011-07-21

**Authors:** Pablo R. Mulieri, Luciano D. Patitucci, Juan A Schnack, Juan C. Mariluis

**Affiliations:** ^1^ Consejo Nacional de Investigaciones Científicas y Técnicas, Buenos Aires, Argentina; ^2^ Administración Nacional de Laboratorios e Institutos de Salud “Dr. Carlos G. Malbrán”, CeNDIE- Departamento Vectores. Av. Vélez Sarsfield 563, 1281, Buenos Aires, Argentina; ^3^ División Entomología, Facultad de Ciencias Naturales y Museo, Universidad Nacional de La Plata, Paseo del bosque s/n°, 1900 La Plata, Buenos Aires, Argentina

**Keywords:** diversity, rural and urban systems, species richness, synanthropy

## Abstract

Sarcophagid species inhabiting different locations in a rural-urban gradient were surveyed in the east central Argentine district of the Almirante Brown, Buenos Aires province. The main objectives of this research were to identify the most prevalent sarcophagid species and to describe community richness and diversity according to the degree of urbanization and the environmental variables measured in three locations within a rural-urban gradient sampled during two years from May 2005 to April 2007. Spatial and seasonal variations were the main factors involved in structuring the sarcophagid communities. Diversity was lower in urbanized areas than in rural ones. Bait and microhabitat preferences (sunny or shady places) and seasonal fluctuations were described for 17 sarcophagid species.

## Introduction

Urban development and its effects on insect species diversity have received increasing attention recently (Blair and Launer 1997; McIntyre et al. 2001; McKinney 2008). This tendency is attributed to the progressive urbanization of natural and agricultural systems (McDonnell et al. 1997). The most remarkable effect of urbanization is the erosion of native biodiversity and, occasionally, species extinctions (Czech et al. 2000). The creation of urban areas is associated with physical changes, such as loss of soil's permeability, air pollution, and the replacement of native species by exotic ones (McDonnell et al. 1997; McKinney 2002).

Changes of biotic communities in urban landscapes are revealed mainly by their structural homogenization. The resulting communities usually show low diversity and remarkable dominance of exotic and, eventually, synanthropic species (McKinney 2002, 2006). The communities generated by human intervention are typical of environmental patches of fragmented, reduced, and isolated habitats (Denys and Schmidt 1998; McIntyre et al. 2001; Gibb and Hochuli 2002).

Calypterate flies have frequently been associated with human settlements and domestic animals. Several worldwide species are attracted by animal feces or carrion and other filthy places, such as garbage disposal sites (Greenberg 1973; Grakzyk et al. 2005). The important role exhibited by synanthopic flies in spreading disease to humans and animals is partially due to their high flight capacity and preference to visit decaying matter and feces (Grakzyk et al. 2005). To estimate the degree of synanthropy (living in association with humans) of fly species, Nuorteva (1963) has proposed a Synanthropy Index (SI). However, the scope of the SI has been generally limited to the assessment of the pest-status of a given species from a sanitary viewpoint. Therefore, this index does not give information on the still poorly known ecological effect of the urbanization process on dipteran communities.

The Sarcophagidae is one of the most diverse families of calypterate flies and includes several saprophagous species. Some substrates, like small vertebrate carcasses, land snails, feces, or horse dung, attract adult sarcophagid flies for feeding or larvipositing (Pape 1998). The Neotropics contains the most diversified sarcophagid fauna (Pape 1996). However, few studies, all restricted to tropical locations, have been conducted to describe temporal and spatial changes in their communities along urban-rural gradients (Ferreira 1979; Linhares 1981; Dias et al. 1984). These authors applied the Synanthropy Index and showed that some species are strongly related to urbanized areas. Several authors reported synanthropic sarcophagids as myasis producers (James 1947; Zumpt 1965; Guimaraes et al. 1983) as well as forensic indicators (Oliva 1997; Byrd and Castner 2001).

Little ecological information is available on sarcophagids from Argentina. Recent works have described the relative abundance, seasonality, and bait and habitat preferences of species occurring in a natural reserve adjacent to Buenos Aires city (Mariluis et al. 2007; Mulieri et al. 2008). Further information on urban taxocoenosis of Sarcophagidae is not currently available from other temperate areas of the country.

This work characterizes and compares sarcophagid assemblages inhabiting different locations along a rural-urban gradient of temperate Argentina, including the identification of the prevalent species and the description of their seasonal and spatial variations, as well as some relevant attributes above population level such as species richness and diversity. Furthermore, the influence of temperature and relative humidity, sunny and shady areas, and the kind of offered baits on the composition of the flesh fly populations were assessed.

## Materials and Methods

### Field sites

**Figure 1.**
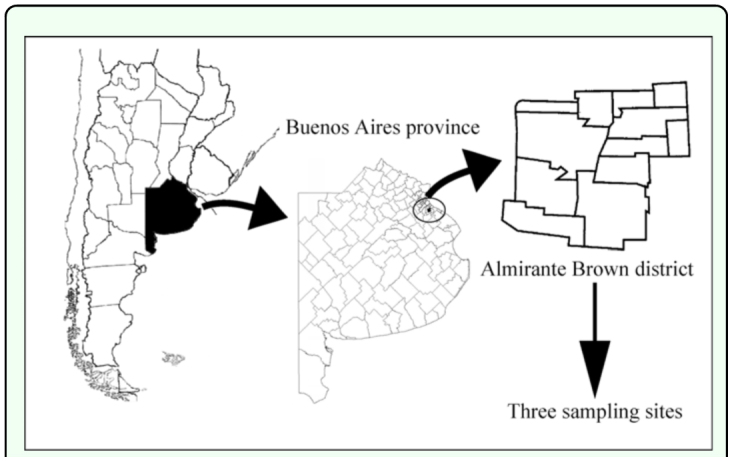
Geographic location of the study site. High quality figures are available online.

The study sites were located in Almirante Brown district, 30 km south Buenos Aires city, Argentina (Figure 1). This district covers 129.33 km^2^ with a population exceeding 0.5 million people. The climate is temperate and humid, with a mean annual precipitation of approximately 1 m.

The three sites selected for this work exhibit different degrees of human intervention along a gradient including urban, suburban, and rural areas, respectively.

The urban site (U) was a densely populated area in the urban core of Burzaco city (34° 50′ 15.02″S, 58° 23′ 52.75″W). This site consisted of buildings and family houses with limited gardens. The U site presented a high proportion of built surface and paved streets. The green areas of this place were frequently subjected to lawn cutting controls.

The sub-urban site (S) was an area of isolated dwellings in the suburbs of Burzaco city (34° 49′ 36.90″S, 58° 24′ 16.56″W). This place exhibited a lower cover of built surface, with small remnant grasslands patches, wooded areas of exotic trees and large gardens.

The rural site (R) was a mix of farm and wild areas (34° 51′ 29.38″S, 58° 23′ 17.75″W), whose vegetation was mainly composed by grasslands patches and native woods, dominated by *Celtis tala* (Ulmaceae) and *Parkinsonia aculeata* (Fabaceae) and dispersed shrubs of *Citrus trifoliata* (Rutaceae) and *Baccharis* spp. (Asteraceae).

### Sampling methods and identification of flies

The samples were taken from May 2005 to April 2007 at monthly intervals. Samples were taken when daily conditions were appropriate (sunny and dry weather). Each month, flies were trapped on attractive baits with a hand net. The baits used were composed of 200 g of rotten cow liver (aged 5 days at ambient temperature) and 200 g of fresh dog feces. For the three sites, liver and feces were simultaneously placed in two points: under the shade of trees (shady condition) and in open pasture or garden (sunny condition), totaling four baits per site. The sampling effort at each site was standardized and consisted of seven hourly capture events (10:00 – 16:00 h) on each bait per date. The baits were exposed for 15 minutes to allow flies to arrive, at the end of which all the arrived flies were captured by net. After each capture the baits were preserved in closed containers until the next capture. All baits were placed on the ground in green areas at the three sites (private gardens in U and S sites and a pasture for R site).

Flies sampled were killed *in situ* by placing them in glass vials with carbon tetrachloride and then stored in paper containers. All the sarcophagid specimens collected were sexed and identified to species. In the case of males, the identification was based on the examination of aedeagal structures. This was performed according to the dissection technique described by Dahlem and Naczi (2006). Other Diptera were identified to family. Taxonomic identifications were performed using specialized literature of local fauna: Sarcophagidae (Mulieri 2008), other Diptera (Shannon and Del Ponte 1926; McAlpine 1981). Voucher specimens are housed in the “Departamento de Vectores”, ANLIS “Dr. Carlos G. Malbrán”.

On each sampling date, climate conditions were characterized by the following variables: mean air temperature and relative humidity. Both variables were recorded during the sampling period (10:00 – 16:00 h) with a digital thermo-hygrometer at hourly intervals.

### Data analysis

Sarcophagid community structure was assessed monthly at each site during the entire study period. The community variables calculated were species richness and the Shannon Diversity Index (Krebs 1999). Abundance was expressed as the number of flies captured per date.

Multiple regression analyses were performed to evaluate the relationships between climatic variables and community parameters (species richness, diversity, or abundance) (Zar 1996). The dependent variables were alternatively abundance, richness, and Shannon diversity of sarcophagid community on each sampling date, and the independent variables were mean temperature and relative humidity. Values of abundance were transformed to *logic*, (*n*+1), and data of relative humidity to *arcsin* √*p*, respectively.

The non-parametric Friedman analysis of variance was used to test for significant differences between the three sites for richness and Shannon diversity, and multiple comparisons were performed according to Tukey procedure (Zar 1996). In addition, rarefaction curve per individuals were used to compare taxonomical diversity in samples of different sizes. The algorithm used is from Krebs (1989). The comparisons of richness, diversity, and abundance between the type of baits or light condition were assessed by means of the non-parametric Wilcoxon test for dependent samples (Zar 1996).

The abundance of sarcophagid species found on each bait type and site was examined by applying Principal Components Analysis (PCA). The PCA describes the pattern of resource use of the species along the gradient in term of few identifiable axes (Nichols 1977). The data matrix for this analysis included original data of 7 variables of resource use (three represented the sites along the gradient, and four represented the different combination of bait type and light level) and 17 dominant species (more than 20 specimens sampled). A variance—covariance matrix was used to obtain the eigenvalues, eigenvectors, and scores of species.

Statistical differences in the occurrence of each species between baits and solar radiation conditions were assessed separately by using the Chi-square test (Zar 1996). Differences in proportional occurrence between sites for each species (*p_sp_* = *n_sp_*/*N_tot.site_*) were assessed by means of test for independent proportions (Fleiss 1981).

## Results

During the sampling period, a high number of specimens of Sarcophagidae was recorded (N = 9,384). However, Calliphoridae was the most abundant family of flies captured (N = 21,865). Other less abundant families were not included in the subsequent analysis; they were Muscidae (N = 3,462), Anthomyiidae (N = 2,197), and Fanniidae (N = 1,969). The remaining collected specimens (Piophilidae, Scatophagidae, Phoridae, Drosophilidae, Tephritidae, Lonchaeidae, and Micropezidae) were very poorly represented, totaling 524 individuals. The overall sarcophagid species richness was 29. Among the sarcophagid species, the adults of both *Oxysarcodexia paulistanensis* (Mattos) and *Tricharaea (Sarcophagula) occidua* (Fabricius) exceeded the number of adults collected for all others species combined (73% of the total number of flies) (Table 1).

**Table 1.**
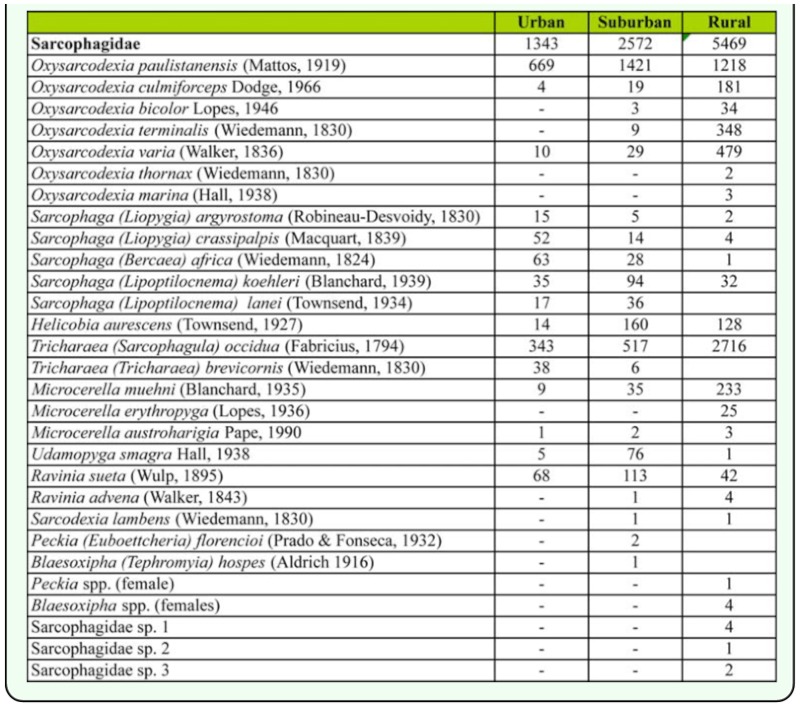
Total abundance of Sarcophagidae collected in the urban-rural gradient, Almirante Brown (Buenos Aires, Argentina), during the study period.

### Seasonality and community structure

**Figure 2.**
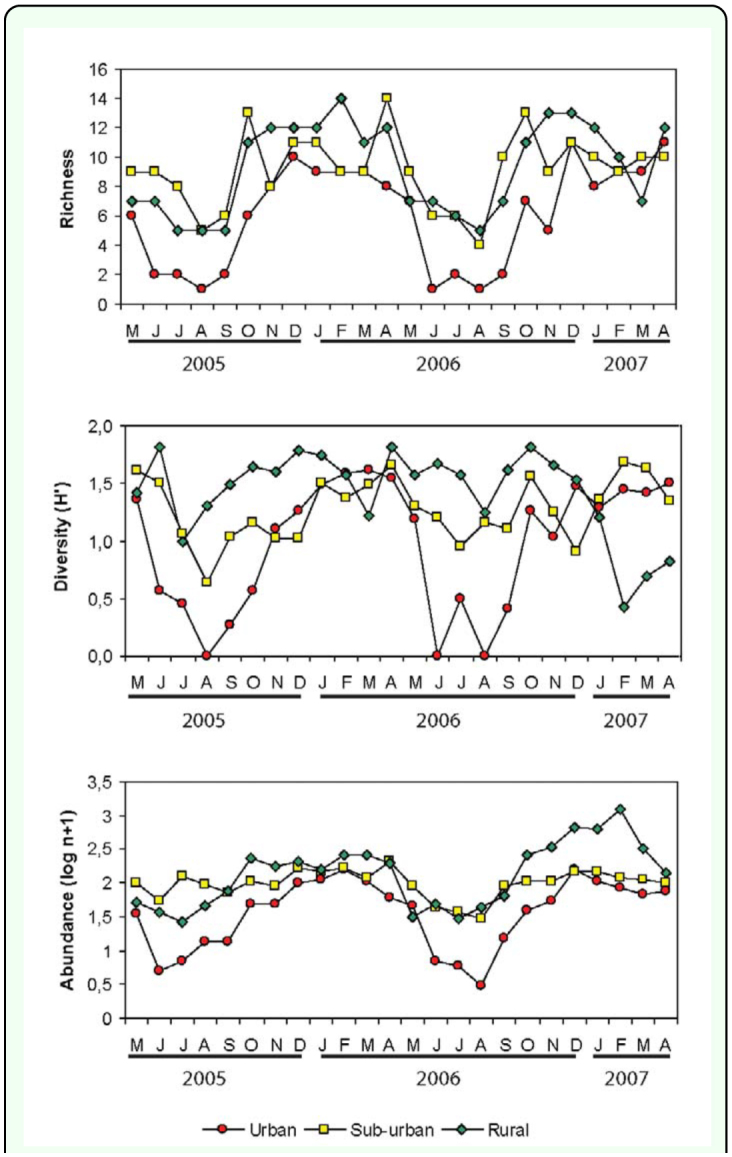
Number of species (richness), diversity (Shannon Diversity Index), and abundance of Sarcophagidae from May 2005 until April 2007, in the urban-rural gradient, Almirante Brown (Buenos Aires, Argentina). High quality figures are available online.

Richness varied throughout the studied period and showed similar fluctuations among the three sites (Figure 2). The highest species richness was recorded between October and April and lowest species richness was recorded between July and August.

**Table 2.**
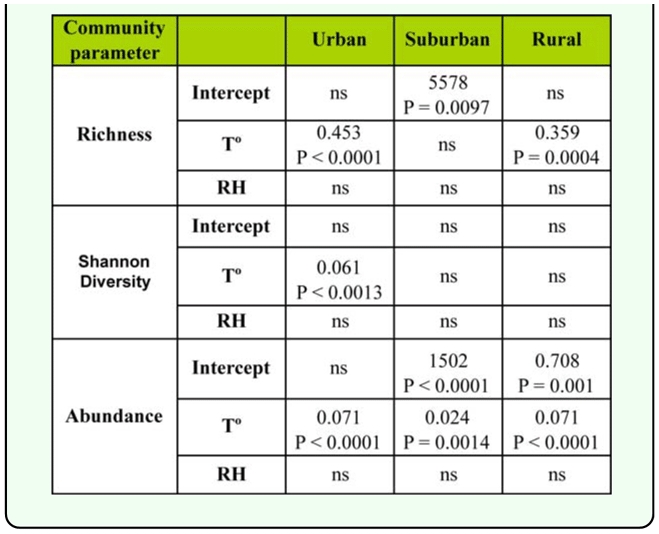
Multiple regression coefficients of meteorological variables (temperature and relative humidity) in relation to the richness, diversity and abundance of Sarcophagidae in the urban-rural gradient, Almirante Brown (Buenos Aires, Argentina).

Multiple regression analysis showed a positive relationship between the richness of Sarcophagidae and mean temperature for the urban site (*r*^2^ = 0.54, *F*_1, 22_ = 26.26, *p* < 0.0001) and rural site (*r*^2^ = 0.45, *F*_1,22_ = 17.66, *p* = 0.0004) (Table 2). Conversely, the suburban site did not show any relationship between species richness and mean temperature (*r*^2^ = 0.19, F_1,22_ = 3.46, *p* = 0.764) (Table 2).

Diversity of Sarcophagidae was recorded at each site differed in their seasonal fluctuation pattern (Figure 2). The urban site exhibited a peak of diversity during February to April (the summer and early autumn) and a drastic reduction of diversity values during July to August (winter). The diversity recorded in both sub-urban and rural sites did not show definite seasonal trends throughout the study period. Multiple regression analysis showed a positive relationship between the diversity and mean temperature only for the urban site (*r*^2^ = 0.38, *F*_1,22_ = 13.65, *p* = 0.0013) (Table 2). The sub-urban (*r*^2^ = 0.05, *F*_1,21_ = 0.531, *p* = 0.595) and rural (*r*^2^ = 0.05, *F*_1,21_ = 0.54, *p* = 0.589) sites did not show significant relationships between this parameter and environmental variables (Table 2).

**Table 3.**
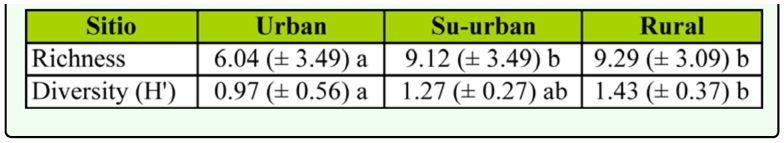
Monthly richness and diversity (mean ± std. dev) of Sarcophagidae in the urban-rural gradient, Almirante Brown (Buenos Aires, Argentina), during the study period. Numbers followed by different letters differ according to multiple comparison test of Tukey.

The abundance of flies captured per sampling date reached its peak between October and April in urban and rural sites (Figure 2), while the sub-urban site did not show remarkable differences throughout the sampling period (Figure 2). A positive relationship between the abundance and mean temperature was recorded for the urban (*r*^2^= 0.61, *F*_1,22_ = 34.28, *p* < 0.0001), sub-urban (*r*^2^ = 0.37, *F*_1,22_ = 13.19, *p* = 0.0015), and rural (*r*^2^ = 0.74, *F*_1,22_ = 61.31, *p* < 0.0001) sites (Table 2).

Relative humidity did not show any relationship with species richness, diversity, and abundance in any of the three study sites (Table 2).

### Diversity along the urban-rural gradient

The richness registered in each site was significantly related to the level of urbanization (Friedman ANOVA χ2_24,2_ = 24.98, *p* < 0.0001). The amount urbanization also affected the diversity of Sarcophagidae (Friedman ANOVA χ2_24,2_ = 11.08, *p* = 0.0039). In both cases, richness and diversity showed highest values in the rural site and decreased in the suburban and urban sites, respectively (Table 3).

**Figure 3.**
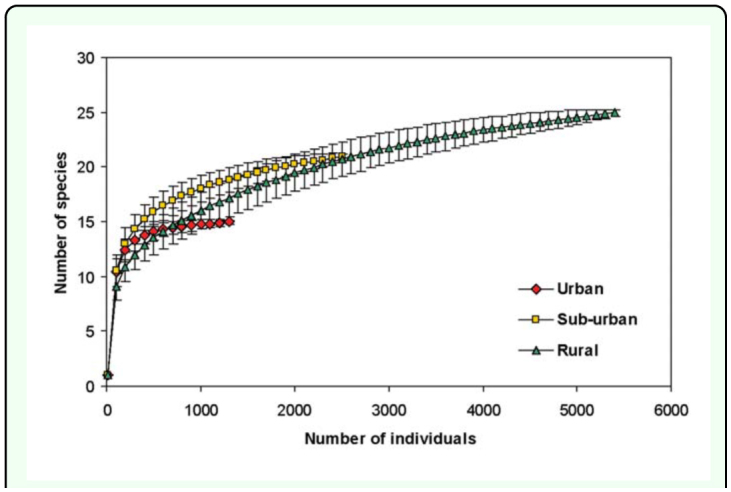
Rarefaction curve for Sarcophagidae in the urban-rural gradient, Almirante Brown (Buenos Aires, Argentina). Bars indicate standard deviation. High quality figures are available online.

The rarefaction curves for the urban, suburban, and rural samples are presented in Figure 3. When rural and suburban samples were rarefied down to 1300 individuals, their species richness exceeded that recorded to urban site. This result indicates that, for an equivalent number of flies, the urban site was poorer in number of species.

**Figure 4.**
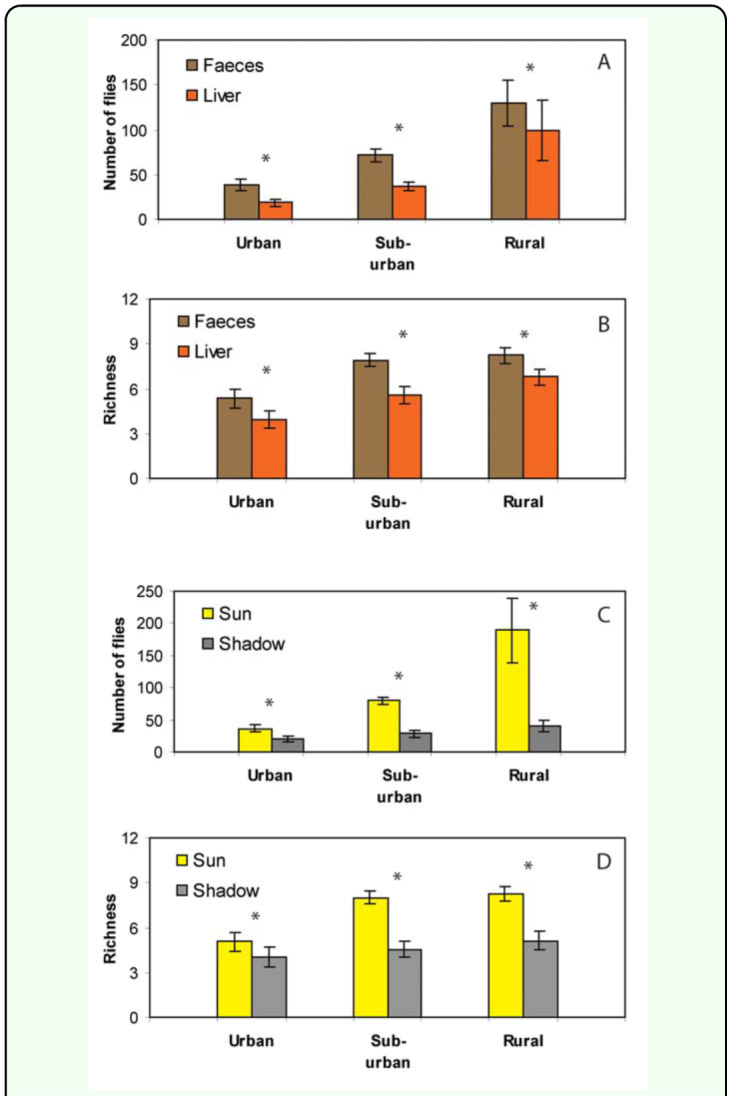
Abundance and richness of Sarcophagidae obtained on different baits. (A) Comparisons of abundance between type of baits. (B) Comparisons of abundance between sunny and shady conditions of baits. (C) Comparisons of richness between types of baits. (D) Comparisons of richness between sunny and shady conditions of baits. Asterisks indicate significant differences (*P* < 0.05) as determined by Wilcoxon test. High quality figures are available online.

The baits used for trapping showed differences in their performance. Sarcophagids were obtained in higher numbers on feces than on rotten cow liver baits in urban (Wilcoxon Pair Test T= 18, *p* = 0.0003), suburban (Wilcoxon Pair Test T = 18, *p* = 0.0002), and rural (Wilcoxon Pair Test T = 57.5, *p* = 0.0082) sites (Figure 4A). The higher attractiveness of feces was also reflected in the higher number of species captured by this kind of bait in the urban (Wilcoxon Pair Test T = 16.5, *p* = 0.0016), suburban (Wilcoxon Pair Test T = 21, *p* = 0.001), and rural (Wilcoxon Pair Test T = 43.5, *p* = 0.0217) sites (Figure 4B).

Microhabitats with different degrees of solar radiation showed significant differences in the three sites. Thus, baits with direct solar incidence attracted a higher number of flies per date than those placed in shady points for the urban (Wilcoxon Pair Test T = 11, *p* = 0.0001), suburban (Wilcoxon Pair Test T = 1, *p* < 0.0001), and rural (Wilcoxon Pair Test T = 1, *p* < 0.0001) sites (Figure 4C). The highest species richness corresponded to baits with direct solar incidence for urban (Wilcoxon Pair Test T = 64, *p* < 0.0424), suburban (Wilcoxon Pair Test T = 5, *p* < 0.0001), and rural (Wilcoxon Pair Test T = 6, *p* < 0.0001) sites (Figure 4D).

### Pattern of resource use and seasonality of the species

**Figure 5.**
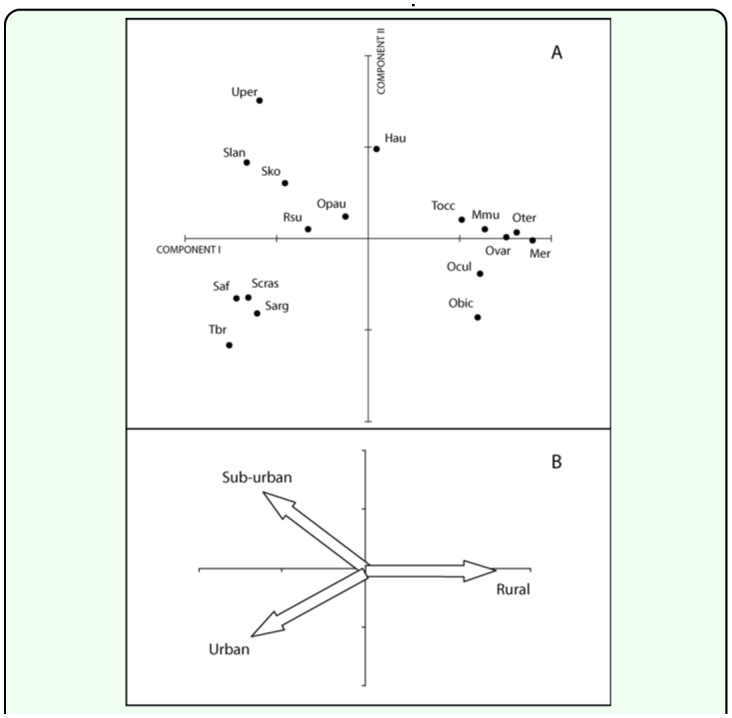
Principal Components Analysis (PCA) ordination graph. (A) Position of sarcophagid species from the urban-rural gradient along first two axes obtained from PCA. (B) Interpretation of the plane defined by the first two components according the most explanatory variables (See Table 4). High quality figures are available online.

Data concerning resource use by dominant sarcophagid species were described by the PCA. Given PCA axes are required to be orthogonal, the third axis is associated with the residual differences among the species, and for this reason the third component was not plotted. Therefore, the first two components can be used to explore variations in species pattern for the more explanatory variables (Figure 5B). The first two components accounted for the 81.0 % of the total variance. The first component was positively associated with the presence of flies in the rural site and negatively associated with both highly and mid-urbanized sites. Therefore, this axis was associated with urbanization degree. The second component accounted for the 19.7 % of variance and differentiated the urban and suburban sites. Secondarily, this second axis differentiated feces baits placed in different conditions of solar radiation (Table 4).

**Table 4.**
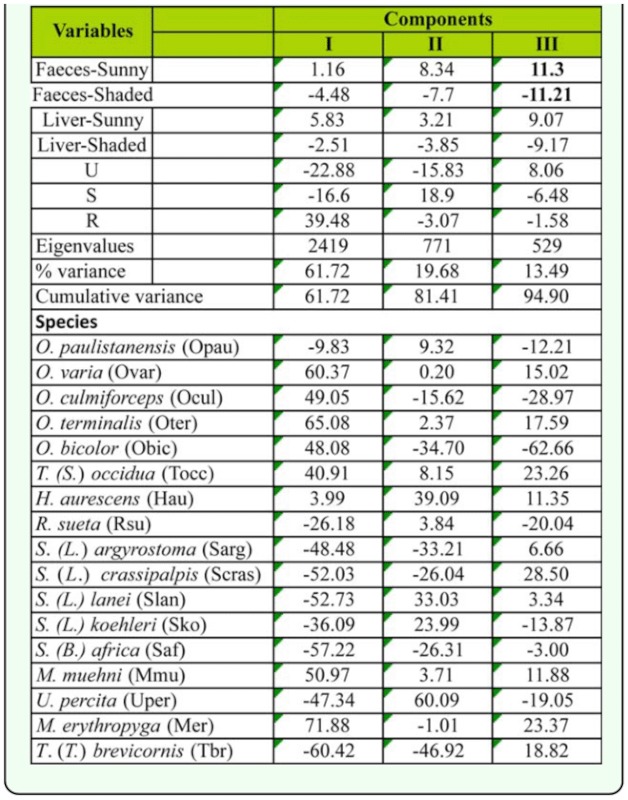
Results of Principal Components Analysis (PCA) based on capture data from Almirante Brown (Buenos Aires, Argentina), during the study period.

In general terms, a lower contribution in the PCA analysis corresponded to variables defined according to the captures on different baits and sunny/shady condition. The relative abundance obtained between baits and between sunny/shady conditions for each species were both compared because of their importance to generate hypothesis regarding the life cycle and microhabitat preference of the species. These results were summarized in Table 5 and 6, where the number of captures on feces, or in sunny sites, were higher than those on liver or shady sites for most species, respectively.

**Table 5.**
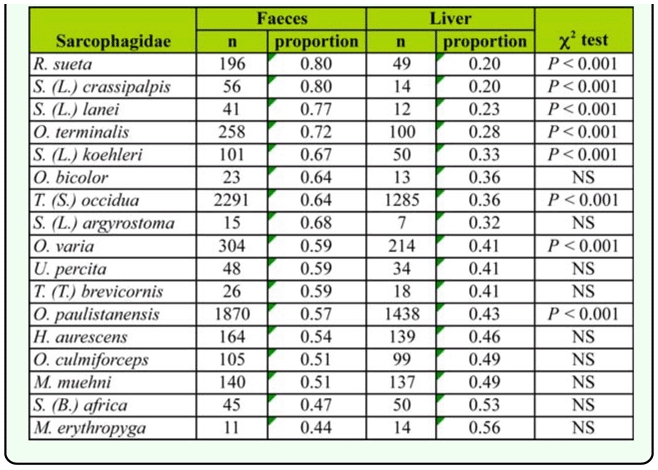
Number and proportion of Sarcophagidae species captured on faeces and liver.

**Table 6.**
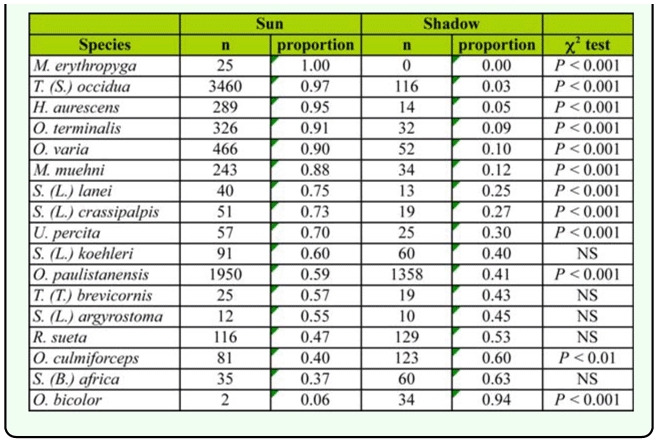
Number and proportion of Sarcophagidae species captured on baits placed in sunny and shady places.

**Figure 6.**
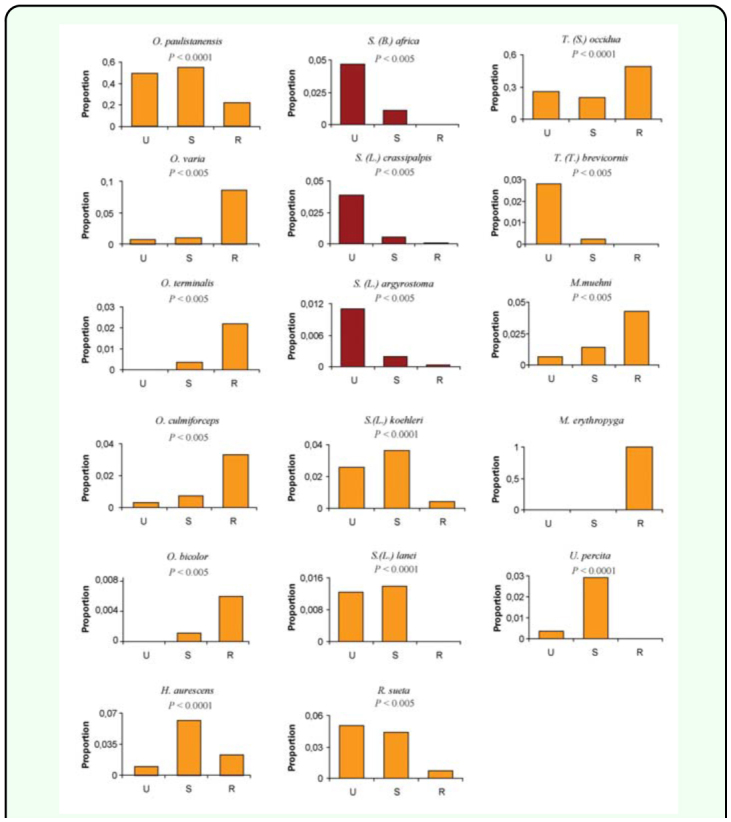
Proportion of Sarcophagidae species to total flies captured for the different sites in the urban-rural gradient during the study period. *P* values account for differences among sites according to the Chi-square test for independent proportions. Orange and red colors represent native and exotic species, respectively. U = urban, S = suburban, R = rural. High quality figures are available online.

The species grouped at the right extreme of the PCA graph were associated with the rural site (Figure 6). Among them, *Oxysarcodexia bicolor* and *O. culmiforceps* were mostly captured on baits placed in the shade (Table 6) and therefore appear in the lower right quadrant of the PCA diagram (Figure 5A). The remaining rural species (*O. varia, O. terminalis, T.* (*S*.) *occidua, Microcerella erythopyga*, and *M. muehni*) were mostly captured on baits placed at sunny locations (Table 6). Conversely, the species placed at the left-lower quadrant of the graph showed increasing captures mostly toward the more urbanized sites (Figure 5A). This group included the three non-native species, *Sarcophaga* (*B*.) *africa, S.* (*L*.) *argyrostoma*, and *S.* (*L*.) *crassipalpis.* Among these synanthropic species, only *S. crassipalpis* showed significantly higher attraction to feces and sunny locations, while the remaining three species did not show statistical differences in their bait resource use (Table 5, Table 6).

Another group of species, at the upper left quadrant of the diagram, included species mostly associated with the suburban site (Figure 5A). However, two species from this group, *O. paulistanensis* and *Ravinia. sueta*, also occurred in high proportions in the urban site (Figure 6), thus occupying an intermediate position in the PCA diagram with respect to the more synanthropic species (Figure 5A). By comparison, the only remaining species, *H. aurescens*, occupied an intermediate position in the diagram, relative to the “rural” species, according to its relative abundance in the rural site (Figure 6).

**Figure 7.**
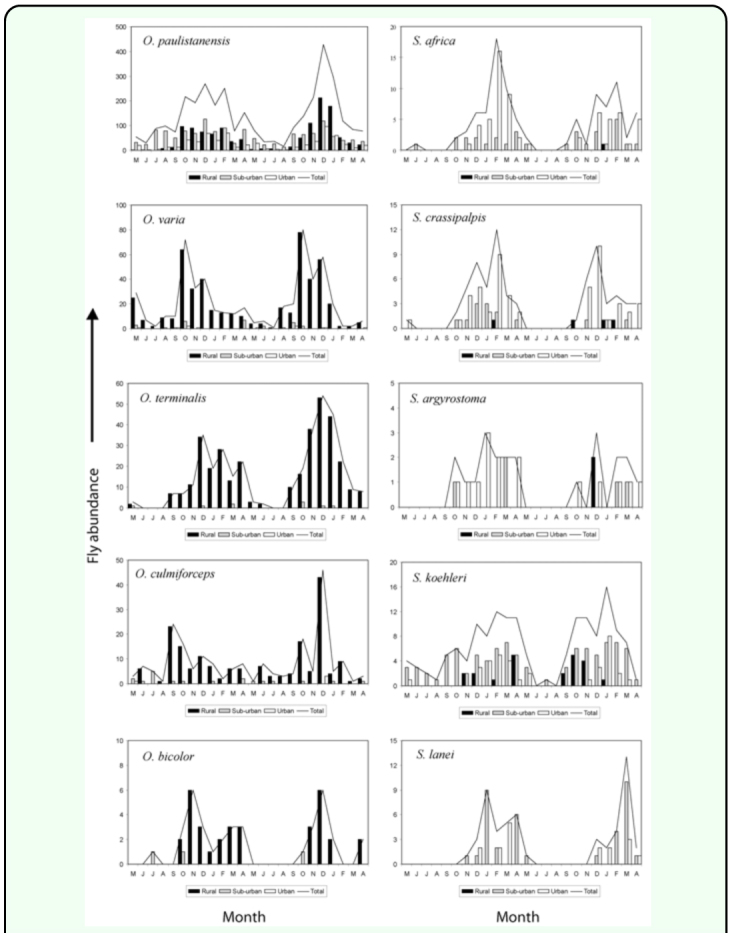
Abundance of *Oxysarcodexia* and *Sarcophaga* species in the urban-rural gradient, Almirante Brown (Buenos Aires, Argentina), during the study period. High quality figures are available online.

**Figure 8.**
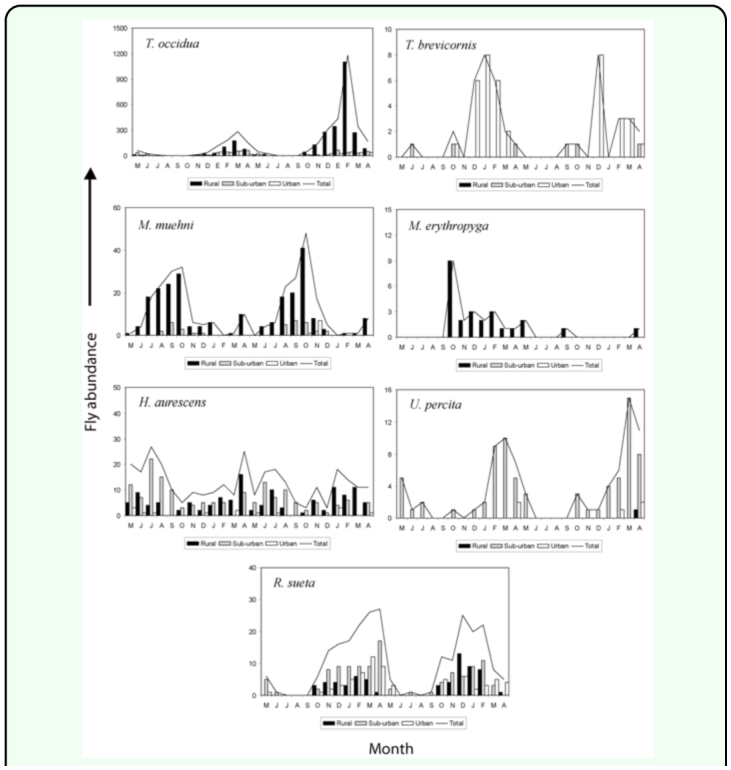
Abundance of *Tricharaea, Microcerella, Helicobia, Udamopyga, Ravinia* species in the urban-rural gradient, Almirante Brown (Buenos Aires, Argentina), during the study period. High quality figures are available online.

Monthly abundance of the dominant sarcophagid species are summarized in the Figures 7 and 8. The synanthropic species (*Tricharaea* (*T*.) *brevicornis, S.* (*B*.) *africa, S.* (*L*.) *argyrostoma*, and S. (*L*.) *crassipalpis*) were noticeably more abundant between December and February (summer period).

Among the species associated with the suburban site, *H. aurescens* occurred predominantly during May to August (autumn and winter), *Udamopyga percita, Sarcophaga* (*L*.) *koehleri*, and *S.* (*L*.) *lanei* did so during December to April, with the latter almost recorded exclusively this time of year.

Among the species of the genus *Oxysarcodexia, O. paulistanensis, O. bicolor*, and *O. varia* occurred in higher numbers between October and March, *O. culmiforceps* was recorded throughout the whole period although in higher numbers in December of the second year, and *O. terminalis* was predominantly captured during November to April (warmer period of the year).

Finally, *T.* (*S*.) *occidua* exhibited a clear seasonal pattern with maximum peaks at the end of summer (February–March), while *M. erythropyga* was recorded during October to May on the first half of the study period.

## Discussion

Urbanization is a process that greatly influences wild sarcophagid communities and usually results in a decrease in species richness. This trend has been recorded in many invertebrate groups, and urbanization frequently fosters the extinction of native species and the colonization of exotic ones (McKinney 2008).

One of the main causes of the local erosion of biodiversity and the extinction of species in urbanized systems is attributed to a species-area effect by loss of habitable area (Faeth and Kane 1978). Typically, urbanization involves the increase of areas covered by pavement and buildings, the removal of native shrubs and trees, and their replacement by grasses and herbs. These changes lead to a drastic isolation of remnant patches of native habitat (McKinney 2002; McDonnell et al. 1997). The above factors may be critical for sarcophagids, and may promote the decrease of larval breeding substrates and the shortage of refuges or sources for obtaining nectar for adults. As observed in the present study, rare species occurred in those sites little affected by urbanization, suggesting that they fail to colonize the isolated and fragmented habitats in urban landscapes.

Some sarcophagids species are able to exploit dead or moribund invertebrates. Among the studied species, *M. muehni* is able to breed on Psychidae and Noctuidae (Lepidoptera) (Blanchard 1939; Blanchard and De Santis 1975), and the species of the genus *Blaesoxipha* Loew act as parasitoid or predators on Coleoptera and Orthoptera (Pape 1994). Specimens of both species were collected almost exclusively at the rural site. This observation suggests that potential prey or dead insects (typically Coleoptera, Lepidoptera, Orthoptera) are not available in dense human settlements. Moreover, previous works showed that urban development may reduce the abundance or richness of Lepidoptera (Blair and Launer 1997) and Coleoptera (Faeth and Kane 1978; Gibbs and Stanton 2001). Depletion of host species might therefore indirectly impact on sarcophagid abundance.

Though urban areas may favor the influx of alien species (McIntyre 2000; McKinney 2006), the addition of these species does not necessarily imply an increase of biodiversity. Three exotic species were recorded, *Sarcophaga argyrostoma, S. crassipalpis*, and *S. africa* in association with urban landscapes. They represent almost 10 % of the total sample for the urban site. Other studies have shown a more drastic replacement of native dipteran by exotic forms (more than 50 % of the total sample), as observed for Drosophilidae and Calliphoridae for urbanized areas of South America (Gottschalk et al. 2007; Mariluis et al. 2008). Nevertheless, previous studies have shown a relative lower prevalence of exotic sarcophagids at urban sites from continental tropical areas as observed in the southeastern Brazilian cities of Campinas (Sao Paulo state) by Linhares (1981) and Belo Horizonte (Minas Gerais state) by Dias et al. (1984).

It is widely speculated that urbanization reduces sarcophagid species richness. Nevertheless, some native species may successfully colonize human-modified environments. Urban and suburban samples were dominated largely by *Oxysarcodexia paulistanensis, Tricharaea occidua*, and *Ravinia sueta*, which represented 80% of the whole sample. These species are coprophilous, and they may act as dung exploiters of domestic animals, especially dog feces, presumably the most common available breeding habitat in the urban landscape.

The seasonal pattern of fly activity, according to the number of species and individuals recorded, was directly related to temperature. Like many insects occupying temperate regions, several sarcophagid species enter in pupal diapause in the coldest season (Denlinger 1978). Peaks of high abundance and species richness were observed during the warmer periods. In addition to the absence of some species during the winter, lower winter activity of adult flies was also detected even for species present all year long, such as *O. paulistanensis.* The lower captures recorded for the colder periods may be attributed to the fact that air temperature may inhibit flying activity and diminish individual developmental rates. This trend was particularly noticeable for the highly synanthropic species, being captured mostly in a short period during the summer (e.g., *Sarcophaga argyrostoma, S. crassipalpis, S. africa, S. lanei*, and *Tricharaea* (s.str.) *brevicornis*).

Because of the saprophagous habits of many sarcophagids species, the baits used in this study represent important breeding substrates or nutritional sources to adult flies. The colonization of baits depends somewhat on the use of the space made by sarcophagid species relative to the site where the dung or carrion is found (Cervenka and Moon 1991; Henning et al. 2005; Mulieri et al. 2008). As reported by other authors, the genera *Tricharaea, Ravinia*, and *Oxysarcodexia* typically include dung—inhabiting species (Mendes and Linhares 1993; O'Hara et al. 1999; Mendes and Linhares 2002). Consequently, the adults of these species are attracted primarily to this kind of substrate (Mendes and Linhares 1993).

Most of the sarcophagid species were captured in higher numbers on baits placed at sunny conditions compared to the baits placed in the shade. Previous studies have reported sarcophagids feeding in areas exposed to high radiation. This is attributed to the fact that their cuticles are of high thermal reflectance. Moreover, they exhibit an active thermoregulatory behavior (Willmer 1982). The only exceptions to this trend were found for *Oxysarcodexia culmiforceps* and *O. bicolor* collected mostly in shady rural microhabitats.

Spatial changes of community structure of sarcophagids are important to know the pattern of resource use of species along the studied gradient. According to their ability to adapt to the conditions existing in the urban-rural gradient and their relationship relative to human activities, the species can be classified into three broad groups: “urban avoiders”, “urban adapters”, and “urban exploiters” (McIntyre 2000; McKinney 2002).

The urban avoiders are the species highly sensitive to human activities. *Microcerella muehni, M. erythropyga*, most species of either the genus *Oxysarcodexia*, or the rare species may be considered within this category.

Urban adapters are typically found in suburban landscapes and include those native species using a wide range of resources, including human-subsidized foods (McKinney 2002). In this study, *Sarcophaga koehleri, S. lanei, H. aurescens*, and *U. percita* were most abundant at the suburban landscape. Although they were not dominant species in the suburban site, *O. paulistanensis, R. sueta*, and *T. occidua* are considered urban adapters due to their high relative abundance in all sites surveyed.

Urban exploiters include those species that almost exclusively exploit food produced by humans (McKinney 2006). Among calypterate dipterans, the species that are limited to the urban site share common traits. They depend on the food produced by the organic matter present in cities. Also, urban exploiters typically include widespread or cosmopolitan species adapted to rocky or areas devoid of vegetation. Among the synanthropic recorded species, *Tricharaea brevicornis* is found on sea beaches (Lopes 1973), and is probably preadapted to the “concrete desert”. On the other hand, *S. africa, S. argyrostoma*, and *S. crassipalpis* are well recognized as cosmopolitan synanthropic species (Povolny and Znojil 1989; Pape 1996, 1998).

Temperate environments have a marked seasonality, which greatly influences the temporal dynamic of sarcophagid assemblages. For instance, *M. muehni* and *H aurecens* showed an exceptional cold tolerance. Accordingly, they may avoid competitive interactions with the remaining “urban avoiders” and “urban adapters” species. However, the temporal resource partitioning is only a possibility and has not been detected with certainty from the data reported.

## Abbreviations

**PCA**,: Principal Component Analysis;
**R**,: rural site;
**S**,: suburban site;
**U**,: urban site
